# Willingness of Patients Prescribed Medications for Lifestyle-Related Diseases to Use Personal Health Records: Questionnaire Study

**DOI:** 10.2196/13866

**Published:** 2020-05-28

**Authors:** Ryoma Hirano, Satoko Yamaguchi, Kayo Waki, Yoshihiko Kimura, Keiichi Chin, Yasuhito Nannya, Masaomi Nangaku, Takashi Kadowaki, Kazuhiko Ohe

**Affiliations:** 1 Nihon Chouzai Co, Ltd Tokyo Japan; 2 Department of Ubiquitous Health Informatics Graduate School of Medicine The University of Tokyo Tokyo Japan; 3 Department of Prevention of Diabetes and Lifestyle-Related Diseases Graduate School of Medicine The University of Tokyo Tokyo Japan; 4 Department of Biomedical Informatics Graduate School of Medicine The University of Tokyo Tokyo Japan; 5 Department of Diabetes and Metabolic Diseases Graduate School of Medicine The University of Tokyo Tokyo Japan; 6 Department of Pathology and Tumor Biology Kyoto University Kyoto Japan; 7 Division of Nephrology and Endocrinology Graduate School of Medicine The University of Tokyo Tokyo Japan

**Keywords:** personal health records, chronic disease, health behavior, eHealth, mHealth, mobile phone

## Abstract

**Background:**

Personal health record (PHR) systems let individuals utilize their own health information to maintain and improve quality of life. Using PHRs is expected to support self-management in patients with lifestyle-related diseases.

**Objective:**

The aim of this study was to identify predictors of the willingness to use PHRs among patients who are prescribed medications for lifestyle-related diseases.

**Methods:**

We recruited pharmacy patrons, aged 20 years or older, who had received at least one medication indicated for hypertension, dyslipidemia, or diabetes. Participants completed self-administered questionnaires regarding their previous diseases, awareness of health care, experience in using PHRs, willingness to use PHRs, and barriers to using PHRs. Data were analyzed using multivariate logistic regression models.

**Results:**

Of the 3708 subjects meeting eligibility criteria, 2307 replies (62.22%) were collected. While only 174 (7.54%) participants had previous PHR experience, 853 (36.97%) expressed willingness to use PHRs. In the multivariate analysis, *considering exercise to be important for health management* (odds ratio [OR] 1.57, 95% CI 1.12-2.21; *P*=.009), *obtaining medical information from books or magazines* (OR 1.23, 95% CI 0.96-1.59; *P*=.10), and *obtaining medical information from the internet* (OR 1.45, 95% CI 1.13-1.87; *P*=.004) were newly identified predictors. These were in addition to known predictors, such as being employed, owning information terminals, and previous PHR experience.

**Conclusions:**

Patients who have an active and positive attitude toward health seem to be more willing to use PHRs. Investigating willingness should contribute to the development of more useful PHRs for self-management among patients prescribed medications for lifestyle-related diseases.

## Introduction

Hypertension, type 2 diabetes, dyslipidemia, and obesity are referred to as lifestyle-related diseases and are major risk factors for coronary heart diseases and strokes if left untreated. The goal of treating lifestyle-related diseases is to maintain good control for a long period of time, staving off mortality. With lifestyle-related diseases, improving diet and exercise and maintaining good medication adherence are crucial [1,2] but doing so for a long period of time can be hard. Adherence to diabetic diet therapy and regular exercise was reported in one study to be 69% and 19%, respectively [3]. For patients with lifestyle-related diseases in general, low medication adherence has been reported [4-6].

Recently, tools that enhance information and communication technology (ICT) have garnered much attention for effectively supporting self-management of lifestyle-related diseases [7]. Prominent in such technology are personal health record (PHR) systems, which consist of information coordination that allows patients to manage and apply their personal health, medical care, and nursing information. The use of PHRs is expected to help prevent progression of lifestyle-related diseases by improving patients’ lifestyles and medication adherence [8,9]. The reported benefits of PHRs were engaging patients in their health behavior, improving their ability to self-manage, and improving communication with health care providers [10,11]. Self-monitoring blood pressure or blood glucose level by using PHRs makes it possible to give patients more specific and direct feedback [10]. A number of PHR systems were developed to support self-management in diabetes patients and were shown to be effective for improving glycemic control [12-14].

We had previously investigated the willingness of diabetes patients to use ICT-based self-management tools and identified predictors of willingness, such as current use of ICT devices or previous dropout from diabetic care [15,16]. However, the role of awareness of health management or medication adherence in this process was not understood, especially among the older population, which accounts for a large percentage of patients with lifestyle-related diseases. Recently, the importance of understanding technology-based behavior in the older population has been emphasized [17,18]. However, little has been understood about willingness and barriers to using PHRs for self-management in the older population. Therefore, we conducted an exploratory study that investigated willingness to use PHRs and awareness of health management in a larger population of patients prescribed medications for lifestyle-related diseases and identified predictors of willingness. Understanding the predictors of willingness to use PHRs may help identify the target population and develop more user-friendly PHRs that could be useful for self-management.

## Methods

### Study Design and Participants

Between February 6 and March 4, 2017, patients who visited any of the 85 Nihon Chouzai pharmacies in Kanagawa Prefecture, Japan, were recruited for the study. Eligibility criteria were being 20 years of age or older and having been prescribed at least one medication typically associated with treatment for hypertension, dyslipidemia, or diabetes (ie, for hypertension, receiving calcium channel blockers, angiotensin II receptor blockers, angiotensin-converting enzyme inhibitors, diuretics, beta adrenergic blockers, or alpha-beta adrenergic blockers; for dyslipidemia, receiving 3-hydroxyl-3-methylglutaryl-CoA reductase inhibitors [ie, statins], fibrate, anion exchange resin, Niemann-Pick C1-Like 1 inhibitor, probucol, nicotinate, or eicosapentaenoic acid; and for diabetes, receiving biguanide, thiazolidine derivatives, sulfonylurea, glinide, dipeptidyl peptidase-4 inhibitor, alfa-glucosidase inhibitor, sodium glucose cotransporter 2 inhibitor, insulin, or glucagon-like peptide-1 receptor agonist [19]). All participants provided written informed consent.

Exclusion criteria were inability to communicate, visiting the pharmacy on behalf of someone else, being judged by pharmacists as inappropriate, receiving in-home treatment, or being admitted to a nursing home.

Of those scheduled to visit the pharmacy during the 4-week study period, 29,771 who met the eligibility criteria were targeted. To assure the collection of questionnaires from 10% of the patients, 5000 were preselected randomly, positing an estimated response rate of 60%. The number of preselected patients at each pharmacy was in proportion to the number of patients who had visited a corresponding pharmacy during the 3 months immediately prior to the study period.

The targeted patients were given both a verbal and written explanation of the study; PHRs were explained both verbally and in writing as “systems such as electronic prescription records or health management apps to manage one’s own medication and health information using electronic devices, including personal computers or smartphones.” Those who responded with written informed consent were asked to complete a self-administered questionnaire. The pharmacists checked questionnaires for completion and collected them.

### Questionnaire Contents

Each questionnaire elicited the following: (1) basic data, including gender, age, and occupation; (2) medical history, including the disease currently being treated, hospitalizations (with time of stay), and diagnosis of metabolic syndrome; (3) awareness of health management, including what patients considered important for maintaining health, their health information sources, the number of regularly prescribed medications, number of doses, their understanding of their diseases, use of pharmacies, and medication adherence; and (4) PHR-related data, including whether they own an information terminal, their use of PHRs, and their willingness to use a PHR (see Multimedia Appendix 1). All information including medical history was self-reported and diagnoses were not validated by medical records.

### Statistical Analyses

The differences between the two groups determined by their willingness to use PHRs were tested for statistical significance using the Fisher exact test. Correlation coefficients between two variables were calculated by the Spearman rank correlation test and were confirmed to be <.8 in all combinations.

The variables were analyzed using univariate logistic regression models. Those variables with an alpha significance level of .10 were considered candidates for explanatory variables in multivariate analyses.

Multivariate logistic regression models were built by stepwise model selection using Akaike’s information criterion. The multivariate analyses included 2020 participants with no missing data in the variables that would comprise the final models.

All statistical analyses were carried out using EZR (Easy R) (Saitama Medical Center, Jichi Medical University, Saitama, Japan) [20].

### Data Exclusion

A few questions on the questionnaire were excluded from the analyses because participants presumably misunderstood them; for example, “use of the family pharmacist system” and “use of Okusuri Techo Plus,” which is an electronic prescription record application for smartphones and personal computers developed by Nihon Chouzai that has various functions, such as sending prescription information to the pharmacy, recording test results, management of medication adherence, and health information distribution.

Although only 20.66% (1033/5000) of the preselected candidates had registered with a family pharmacist, 43.43% (1002/2307) of the participants responded that they used the family pharmacist system. Similarly, although the actual usage ratio of Okusuri Techo Plus stood at only 5.92% (296/5000) among preselected candidates, 45.08% (1040/2307) answered that they used it.

## Results

### Study Population

**Figure 1:**
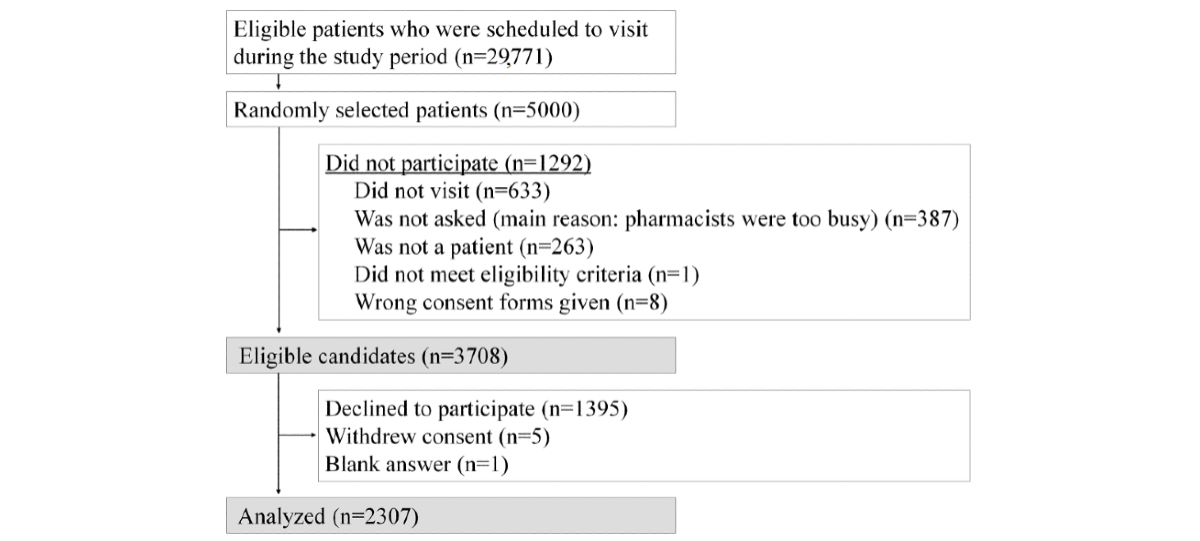
Flowchart of participants in this study. Random selection from a population of patients prescribed medications for lifestyle-related diseases, confirmation of eligibility, informed consent, and questionnaire collection.

As described in the Methods section, 5000 patients were preselected. A total of 633 people did not visit the pharmacies with preselected patients’ prescriptions during the study period. In total, 387 people were not given an explanation about the study, mainly because pharmacists were too busy, and 263 were excluded because they came to collect someone else’s medication. Among the patients who gave written informed consent, 9 were excluded: 1 was not of age and 8 were given the wrong consent forms by their pharmacists. Overall, 3708 were considered eligible candidates. In total, 1395 declined to participate, 5 withdrew from the study before completing the questionnaire, and 1 turned in a blank answer form, leaving a final number of 2307 valid responses. That made the valid response rate 62.22% (2307/3708) (see Figure 1).

As shown in Table 1, out of 2307 respondents, 1269 (55.01%) were male, 1636 (70.91%) were 60 years of age or older, and 924 (40.05%) were employed; 1345 (58.30%) reported they had hypertension, 705 (30.56%) had diabetes, and 432 (18.73%) had dyslipidemia. Out of 2307 respondents, 345 (14.95%) who reported neither hypertension, diabetes, nor dyslipidemia were included in the analysis, though some of them may have been treated for other diseases using the same medications used to treat hypertension, diabetes, and hyperlipidemia. Out of 2307 respondents, 1302 (56.44%) owned either a personal computer, smartphone, or tablet, with 899 out of 2307 (38.97%) having a smartphone. Out of 2307 respondents, 174 (7.54%) answered that they had experience using a PHR and 853 (36.97%) expressed willingness to use a PHR.

**Table 1:** Univariate models of willingness to use personal health records (PHRs).

Characteristic		Total (N=2307), n (%)^a^	Willingness to use PHRs (n=2155), n (%)^b^		Odds ratio (95% CI)		*P* value	
			Yes (n=853)	No (n=1302)				
**Gender**								
	Male	1269 (55.01)	548 (64.2)	648 (49.77)		1.81 (1.51-2.17)		<.001
	Female	1007 (43.65)	298 (34.9)	637 (48.92)		1.00		
**Age (years)**								
	≥60	1636 (70.91)	488 (57.2)	1017 (78.11)		0.36 (0.30-0.44)		<.001
	<60	654 (28.35)	363 (42.6)	274 (21.04)		1.00		
**Employment status**								
	Employed	924 (40.05)	468 (54.9)	414 (31.80)		2.57 (2.15-3.09)		<.001
	Not employed	1352 (58.60)	380 (44.5)	866 (66.51)		1.00		
**Medical history**								
	Hypertension	1345 (58.30)	471 (55.2)	782 (60.06)		0.81 (0.67-0.97)		.02
	Diabetes	705 (30.56)	276 (32.4)	382 (29.34)		1.15 (0.95-1.39)		.16
	Dyslipidemia	432 (18.73)	191 (22.4)	225 (17.28)		1.37 (1.10-1.72)		.004
	Angina pectoris or myocardial infarction	265 (11.49)	91 (10.7)	150 (11.52)		0.91 (0.68-1.21)		.53
	Arrhythmia	205 (8.89)	77 (9.0)	115 (8.83)		1.02 (0.74-1.39)		.94
	Kidney disease	142 (6.16)	52 (6.1)	78 (5.99)		1.01 (0.69-1.48)		>.99
	Hyperuricemia	125 (5.42)	59 (6.9)	64 (4.92)		1.43 (0.98-2.10)		.06
	Cerebrovascular disorder	132 (5.72)	40 (4.7)	81 (6.22)		0.74 (0.49-1.10)		.13
**Hospitalization experience**								
	Yes	1426 (61.81)	515 (60.4)	814 (62.52)		0.86 (0.71-1.03)		.10
	No	796 (34.50)	321 (37.6)	436 (33.49)		1.00		
**Metabolic syndrome**								
	Yes	543 (23.54)	258 (30.2)	257 (19.74)		1.73 (1.40-2.13)		<.001
	No	1506 (65.28)	514 (60.3)	886 (68.05)		1.00		
	Unknown	239 (10.36)	78 (9.1)	145 (11.14)		0.93 (0.68-1.26)		
**What patients considered important for their health management**								
	Exercise	1975 (85.61)	774 (90.7)	1081 (83.03)		1.98 (1.50-2.64)		<.001
	Diet	2066 (89.55)	795 (93.2)	1146 (88.02)		1.84 (1.33-2.57)		<.001
	Sleep	1593 (69.05)	628 (73.6)	867 (66.59)		1.39 (1.15-1.70)		<.001
	Prescription drugs	1188 (51.50)	431 (50.5)	672 (51.61)		0.95 (0.80-1.14)		.60
	Over-the-counter drugs	44 (1.91)	14 (1.6)	29 (2.23)		0.73 (0.35-1.44)		.43
	Health food and supplements	200 (8.67)	64 (7.5)	121 (9.29)		0.79 (0.57-1.09)		.16
**What patients were willing to incorporate into daily life in managing health**								
	Exercise	1451 (62.90)	554 (64.9)	795 (61.06)		1.19 (0.99-1.43)		.06
	Healthy diet	1907 (82.66)	714 (83.7)	1071 (82.26)		1.12 (0.88-1.42)		.35
	Sleep	1272 (55.14)	479 (56.2)	711 (54.61)		1.07 (0.89-1.28)		.48
	Prescription drugs	1342 (58.17)	505 (59.2)	745 (57.22)		1.09 (0.91-1.30)		.35
	Over-the-counter drugs	38 (1.65)	16 (1.9)	18 (1.38)		1.37 (0.65-2.85)		.38
	Health food and supplements	254 (11.01)	94 (11.0)	145 (11.14)		0.99 (0.74-1.31)		>.99
**Health information sources**								
	Doctors	1939 (84.05)	742 (87.0)	1078 (82.80)		1.37 (1.06-1.77)		.01
	Nurses	208 (9.02)	80 (9.4)	116 (8.91)		1.06 (0.77-1.44)		.76
	Pharmacists	398 (17.25)	176 (20.6)	197 (15.13)		1.45 (1.15-1.83)		.001
	Dieticians	97 (4.20)	45 (5.3)	48 (3.69)		1.45 (0.93-2.25)		.08
	Acquaintances	255 (11.05)	98 (11.5)	143 (10.98)		1.05 (0.79-1.39)		.73
	Family	311 (13.48)	128 (15.0)	164 (12.60)		1.22 (0.94-1.58)		.12
	Books or magazines	454 (19.68)	194 (22.7)	233 (17.90)		1.35 (1.08-1.68)		.007
	Newspapers	450 (19.51)	161 (18.9)	261 (20.05)		0.93 (0.74-1.16)		.51
	Television	836 (36.24)	306 (35.9)	475 (36.48)		0.97 (0.81-1.17)		.75
	Internet	722 (31.30)	432 (50.6)	266 (20.43)		3.98 (3.28-4.84)		<.001
**Understanding of one’s diseases**								
	Understanding one’s diseases or symptoms well	2055 (89.08)	808 (94.7)	1137 (87.33)		2.61 (1.83-3.79)		<.001
	Understanding treatment methods well	1472 (63.81)	592 (69.4)	805 (61.83)		1.39 (1.15-1.68)		<.001
	Understanding how to deal with deterioration of health	591 (25.62)	249 (29.2)	312 (23.96)		1.30 (1.07-1.59)		.009
	Entrusting doctors with disease and health-related matters	910 (39.45)	312 (36.6)	539 (41.40)		0.81 (0.68-0.97)		.02
	Not paying much attention to one’s disease	130 (5.64)	33 (3.9)	82 (6.30)		0.60 (0.38-0.91)		.01
**Information terminals**								
	Personal computer	958 (41.53)	526 (61.7)	404 (31.03)		3.54 (2.94-4.27)		<.001
	Smartphone	899 (38.97)	522 (61.2)	354 (27.19)		4.19 (3.47-5.07)		<.001
	Basic cell phone	687 (29.78)	215 (25.2)	447 (34.33)		0.63 (0.52-0.77)		<.001
	Tablet device	296 (12.83)	190 (22.3)	99 (7.60)		3.44 (2.63-4.51)		<.001
**Number of regularly prescribed medications**								
	≥3	1670 (72.39)	592 (69.4)	969 (74.42)		0.75 (0.61-0.91)		.003
	<3	597 (25.88)	258 (30.2)	315 (24.19)		1.00		
**Number of doses per day**								
	≥2	1460 (63.29)	520 (61.0)	848 (65.13)		0.80 (0.67-0.96)		.02
	<2	784 (33.98)	324 (38.0)	423 (32.49)		1.00		
**Monthly visit frequency to a pharmacy**								
	≥2	419 (18.16)	160 (18.8)	225 (17.28)		1.10 (0.87-1.38)		.42
	<2	1844 (79.93)	686 (80.4)	1060 (81.41)		1.00		
**Medication adherence**								
	Good	1762 (76.38)	638 (74.8)	1032 (79.26)		0.76 (0.61-0.94)		.01
	Poor	506 (21.93)	209 (24.5)	257 (19.74)		1.00		
**Experience using a PHR**								
	Yes	174 (7.54)	164 (19.2)	10 (0.77)		30.30 (15.92-64.79)		<.001
	No	2059 (89.25)	686 (80.4)	1269 (97.47)		1.00		

^a^Percentages are out of 2307; because of missing responses, the percentages may not add up to 100%.

^b^Percentages are out of 853 (for *Yes*) or 1302 (for *No*); because of missing responses, the percentages may not add up to 100%.

### Univariate Analysis

Factors associated with willingness to use PHRs were identified by univariate analysis (see Table 1). Among the basic characteristics, being male (odds ratio [OR] 1.81, 95% CI 1.51-2.17; *P*<.001), and being employed (OR 2.57, 95% CI 2.15-3.09; *P*<.001) were positive predictive factors, while being 60 years of age or older (OR 0.36, 95% CI 0.30-0.44; *P*<.001) was a negative predictive factor.

Regarding medical history, having dyslipidemia (OR 1.37, 95% CI 1.10-1.72; *P*=.004) and having been diagnosed with metabolic syndrome (OR 1.73, 95% CI 1.40-2.13; *P*<.001) were positive predictive factors, while having hypertension (OR 0.81, 95% CI 0.67-0.97; *P*=.02) was a negative predictive factor.

Regarding what patients considered important for their health management, positive predictive factors were exercise (OR 1.98, 95% CI 1.50-2.64; *P*<.001), diet (OR 1.84, 95% CI 1.33-2.57; *P*<.001), and sleep (OR 1.39, 95% CI 1.15-1.70; *P*<.001). Other positive predictive factors were sources of medical information, such as doctors (OR 1.37, 95% CI 1.06-1.77; *P*=.01), pharmacists (OR 1.45, 95% CI 1.15-1.83; *P*=.001), books or magazines (OR 1.35, 95% CI 1.08-1.68; *P*=.007), and the internet (OR 3.98, 95% CI 3.28-4.84; *P*<.001), while nurses, dieticians, acquaintances, family, newspapers, and television were not. Negative predictive factors were *regularly taking three or more types of medicine* (OR 0.75, 95% CI 0.61-0.91; *P*=.003), *taking medicine twice or more per day* (OR 0.80, 95% CI 0.67-0.96; *P*=.02), and *good adherence to taking medicine* (OR 0.76, 95% CI 0.61-0.94; *P*=.01).

Regarding understanding of diseases, positive predictive factors were *understanding one’s diseases or symptoms well* (OR 2.61, 95% CI 1.83-3.79; *P*<.001), *understanding treatment methods well* (OR 1.39, 95% CI 1.15-1.68; *P*<.001), and *understanding how to deal with deterioration of health* (OR 1.30, 95% CI 1.07-1.59; *P*=.009). In contrast, *entrusting doctors with disease and health-related matters* (OR 0.81, 95% CI 0.68-0.97; *P*=.02) or *not paying much attention to one's disease* (OR 0.60, 95% CI 0.38-0.91; *P*=.01) were negative predictive factors.

Regarding PHR-related factors, owning a personal computer (OR 3.54, 95% CI 2.94-4.27; *P*<.001), smartphone (OR 4.19, 95% CI 3.47-5.07; *P*<.001), or tablet device (OR 3.44, 95% CI 2.63-4.51; *P*<.001) were positive predictors, while owning a basic cell phone (OR 0.63, 95% CI 0.52-0.77; *P*<.001) was a negative predictor. Having experience using a PHR (OR 30.30, 95% CI 15.92-64.79; *P*<.001) was a strong positive predictive factor.

### Multivariate Logistic Regression Analyses

Multivariate logistic regression analyses were performed. The final model was identified as male (OR 1.52, 95% CI 1.22-1.90; *P*<.001), employed (OR 1.30, 95% CI 1.03-1.64; *P*=.03); having dyslipidemia (OR 1.25, 95% CI 0.97-1.62; *P*=.09); considering exercise important for health management (OR 1.57, 95% CI 1.12-2.21; *P*=.009); obtaining medical information from doctors (OR 1.36, 95% CI 1.01-1.83; *P*=.04), pharmacists (OR 1.63, 95% CI 1.24-2.13; *P*<.001), books or magazines (OR 1.23, 95% CI 0.96-1.59; *P*=.10), or the internet (OR 1.45, 95% CI 1.13-1.87; *P*=.004); understanding one’s diseases or symptoms well (OR 1.37, 95% CI 0.92-2.05; *P*=.13); entrusting doctors with disease and health-related matters (OR 1.21, 95% CI 0.98-1.51; *P*=.08); owning a personal computer (OR 1.85, 95% CI 1.46-2.35; *P*<.001), smartphone (OR 2.04, 95% CI 1.61-2.59; *P*<.001), or tablet device (OR 1.36, 95% CI 0.99-1.86; *P*=.06); and having experience using a PHR (OR 16.60, 95% CI 8.52-32.20; *P*<.001). All these were positive predictors for the willingness to use a PHR (see Table 2).

**Table 2:** Univariate and multivariate logistic models of willingness to use personal health records (PHRs).

Characteristic		Univariate model (N=2307)				Multivariate model (n=2020)			
		Odds ratio (95% CI)		*P* value		Odds ratio (95% CI)		*P* value	
(Intercept)						0.06 (0.03-0.10)		<.001	
Gender (male vs female)		1.81 (1.51-2.16)		<.001		1.52 (1.22-1.90)		<.001	
Age (≥60 years vs <60)		0.36 (0.30-0.44)		<.001		N/A^a^			
Employment status (employed vs not employed)		2.58 (2.15-3.08)		<.001		1.30 (1.03-1.64)		.03	
**Medical history (yes vs no)**									
	Hypertension		0.81 (0.68-0.96)		.02		N/A^a^		
	Diabetes		1.15 (0.95-1.38)		.15		N/A^b^		
	Dyslipidemia		1.38 (1.11-1.71)		.004		1.25 (0.97-1.62)		.09
	Angina pectoris or myocardial infarction		0.91 (0.69-1.20)		.52		N/A^b^		
	Arrhythmia		1.02 (0.75-1.38)		.90		N/A^b^		
	Kidney disease		1.01 (0.71-1.46)		.94		N/A^b^		
	Hyperuricemia		1.43 (0.99-2.06)		.054		N/A^a^		
	Cerebrovascular disorder		0.74 (0.50-1.09)		.13		N/A^b^		
	Hospitalization experience		0.86 (0.72-1.03)		.10		N/A^b^		
	Metabolic syndrome		1.73 (1.41-2.12)		<.001		N/A^a^		
**What patients considered important for their health management (yes vs no)**									
	Exercise		1.98 (1.51-2.61)		<.001		1.57 (1.12-2.21)		.009
	Diet		1.84 (1.34-2.52)		<.001		N/A^a^		
	Sleep		1.39 (1.15-1.69)		<.001		N/A^a^		
	Prescription drugs		0.95 (0.80-1.13)		.60		N/A^b^		
	Over-the-counter drugs		0.73 (0.38-1.39)		.34		N/A^b^		
	Health food and supplements		0.79 (0.58-1.08)		.15		N/A^b^		
**What patients were willing to incorporate into daily life in managing health (yes vs no)**									
	Exercise		1.19 (0.99-1.42)		.06		N/A^a^		
	Diet		1.12 (0.89-1.41)		.34		N/A^b^		
	Sleep		1.07 (0.90-1.27)		.46		N/A^b^		
	Prescription drugs		1.09 (0.91-1.30)		.34		N/A^b^		
	Over-the-counter drugs		1.37 (0.69-2.69)		.37		N/A^b^		
	Health food and supplements		0.99 (0.75-1.30)		.94		N/A^b^		
**Health information sources (yes vs no)**									
	Doctors		1.37 (1.07-1.75)		.01		1.36 (1.01-1.83)		.04
	Nurses		1.06 (0.78-1.42)		.72		N/A^b^		
	Pharmacists		1.45 (1.16-1.82)		.001		1.63 (1.24-2.13)		<.001
	Dieticians		1.45 (0.96-2.20)		.08		N/A^a^		
	Acquaintances		1.05 (0.80-1.38)		.73		N/A^b^		
	Family		1.22 (0.95-1.57)		.12		N/A^b^		
	Books or magazines		1.35 (1.09-1.67)		.006		1.23 (0.96-1.59)		.10
	Newspapers		0.93 (0.74-1.15)		.49		N/A^b^		
	Television		0.97 (0.81-1.16)		.74		N/A^b^		
	Internet		3.98 (3.29-4.82)		<.001		1.45 (1.13-1.87)		.004
**Understanding of one’s diseases (yes vs no)**									
	Understanding one’s diseases or symptoms well		2.61 (1.84-3.70)		<.001		1.37 (0.92-2.05)		.13
	Understanding treatment methods well		1.39 (1.16-1.67)		<.001		N/A^a^		
	Understanding how to deal with deterioration of health		1.30 (1.07-1.58)		.008		N/A^a^		
	Entrusting doctors with disease and health-related matters		0.81 (0.68-0.97)		.02		1.21 (0.98-1.51)		.08
	Not paying much attention to one’s disease		0.60 (0.40-0.90)		.01		N/A^a^		
**Information terminals (yes vs no)**									
	Personal computer		3.54 (2.95-4.25)		<.001		1.85 (1.46-2.35)		<.001
	Smartphone		4.19 (3.48-5.04)		<.001		2.04 (1.61-2.59)		<.001
	Basic cell phone		0.63 (0.52-0.77)		<.001		N/A^a^		
	Tablet device		3.44 (2.65-4.47)		<.001		1.36 (0.99-1.86)		.06
Number of regularly prescribed medications (≥3 vs <3)		0.75 (0.62-0.91)		.003		N/A^a^			
Number of doses per day (≥2 vs <2)		0.80 (0.67-0.96)		.02		N/A^a^			
Monthly visit frequency to a pharmacy (≥2 vs <2)		1.10 (0.88-1.38)		.41		N/A^b^			
Medication adherence (good vs poor)		0.76 (0.62-0.94)		.010		N/A^a^			
Experience using a PHR (yes vs no)		30.30 (15.90-57.80)		<.001		16.60 (8.52-32.20)		<.001	

^a^N/A: not applicable; variables were considered candidates in the multivariate analyses (*P*<.10) but were discarded from the final model.

^b^N/A: not applicable; variables were not considered candidates for explanatory variables in multivariate analyses (*P*>.10).

### Experience and Willingness to Use Personal Health Records

Participants who answered that they had used a PHR (174/2307, 7.54%) were asked the following question about their experience: Which applications had they used? The most common responses were *prescription records* (129/174, 74.1%), *activity amount recorders* (43/174, 24.7%), *blood pressure management* (37/174, 21.3%), and *weight management* (27/174, 15.5%) (see Table 3).

**Table 3:** Type of personal health record (PHR) application used by participants.

Type of application	Participants (n=174), n (%)
Prescription records	129 (74.1)
Activity amount recorders	43 (24.7)
Blood pressure management	37 (21.3)
Weight management	27 (15.5)
Information on sleep	13 (7.5)
Glycemic control	10 (5.7)
Menstrual management	4 (2.3)
Others	3 (1.7)

The types of information that respondents had most commonly managed through the use of a PHR included *medication prescription details* (76/174, 43.7%), *medication adherence* (54/174, 31.0%), and *home blood pressure values and body weight, etc* (50/174, 28.7%). When asked what types of information they wanted to manage, many responded with answers related to medication, such as *medication prescription details* (84/174, 48.3%) and *medication adherence* (75/174, 43.1%), as well as those related to test values, such as *test results at medical institution* (82/174, 47.1%) and *home blood pressure values and body weight, etc* (76/174, 43.7%) (see Table 4). It was found that while many people expressed a desire to manage test results at medical institutes using a PHR, they were not actually doing so (82/174, 47.1%, vs 20/174, 11.5%; *P*<.001, Fisher exact test). We also found a gap between participants’ expressed desires and reality as to *home blood pressure values and body weight etc*, *medication adherence*, *record of the date of hospital visit*, *medical history*, and *meal content* (see Table 4). The most common sources of information about PHRs were *pharmacy staff* (107/174, 61.5%), followed by *the internet* (53/174, 30.5%) (see Table 5).

**Table 4:** Type of information that personal health record (PHR) users wanted to manage, or did manage, by PHR.

Information type	Participants (n=174), n (%)		*P* value
	Wanted to manage	Managed	
Medication prescription details	84 (48.3)	76 (43.7)	.45
Test results at medical institution	82 (47.1)	20 (11.5)	<.001
Home blood pressure values and body weight, etc	76 (43.7)	50 (28.7)	.005
Medication adherence	75 (43.1)	54 (31.0)	.03
Record of the date of hospital visit	40 (23.0)	21 (12.1)	.01
Medical history	35 (20.1)	8 (4.6)	<.001
Meal content	24 (13.8)	9 (5.2)	.010
Record of immunization	10 (5.7)	3 (1.7)	.09
Surgery record	6 (3.4)	2 (1.1)	.28
Medical history of the family	4 (2.3)	2 (1.1)	.68
Others	3 (1.7)	3 (1.7)	>.99

**Table 5:** Sources of information about personal health records (PHRs).

Information source^a^	Participants (n=174), n (%)
Pharmacy staff	107 (61.5)
Internet	53 (30.5)
Family	13 (7.5)
Poster	8 (4.6)
Television	6 (3.4)
Hospital staff	5 (2.9)
Newspapers	2 (1.1)
Books or magazines	2 (1.1)
Acquaintance	1 (0.6)
Others	8 (4.6)

^a^Participants who had previous PHR experience were asked where they got information from; multiple answers were allowed.

The 1302 participants who expressed unwillingness to use PHRs were asked why. The main reasons were *it needs more time and effort* (509/1302, 39.09%) or *the need is not felt* (465/1302, 35.71%), followed by *concern over security* (199/1302, 15.28%) (see Table 6).

**Table 6:** Reasons for unwillingness to use a personal health record (PHR).

Reasons^a^	Participants (n=1302), n (%)
More time and effort	509 (39.09)
The need is not felt	465 (35.71)
Concern over security	199 (15.28)
Cannot use mobile devices	35 (2.69)
Have no mobile device	17 (1.31)
Other	86 (6.61)
No answer	131 (10.06)

^a^Respondents who expressed unwillingness to use PHRs were asked the reasons why; multiple answers were allowed.

## Discussion

### Principal Findings

Among the predictive factors identified in this study, owning a personal computer, smartphone, or tablet and having experience using a PHR were consistent with prior reports that ICT literacy is associated with willingness to use a PHR [15,16,21-23]. While older age was reported to be a negative predictor in some studies [24,25], others reported that current use of ICT devices was more associated with willingness than age [21,23]. Consistently, in this study, although univariate analysis showed that older age was a negative predictor of willingness to use a PHR (see Table 1), that factor was discarded from the final model of the multivariate analysis (see Table 2). The ownership rate of personal computers, smartphones, and/or tablets was 88.7% (580/654) among participants under 60 years of age, but it was only 43.89% (718/1636) among participants 60 years of age or older (see Multimedia Appendix 2), clearly demonstrating the digital divide between generations. While 55.5% (363/654) of the participants under 60 years of age expressed willingness, only 29.83% (488/1636) of those 60 years of age or older did so (see Table 1). However, among participants 60 years of age or older, 48.1% (345/718) of those who owned ICT devices expressed willingness to use PHRs, compared to only 16.4% (136/831) of those who did not own these devices (see Multimedia Appendix 2). Being employed was identified as a predictor of willingness to use PHRs in this study, consistent with the previous reports [15,26].

While most of the previous reports suggested that gender was not a predictor [21,22,25,27], being male was identified as a predictor in this study. Consistently, the users of GlucoNote—an app for type 2 diabetes and prediabetes patients that we recently developed and released—were mostly men [28]. In contrast, studies in the United States and Europe reported that being female was a predictor of electronic health (eHealth) use [26,29]. These differences in results may be explained, at least partly, by different gender roles in each country; it was reported that a persistent gender gap in ICT use and skills was observed in Japan but not in the United States after adjusting for employment status [30].

The finding that participants who consider exercise important for their health management are more willing to use PHRs (see Table 2) was novel, as was the finding that patients who obtain medical information from doctors, pharmacists, books or magazines, and the internet are more willing to use PHRs. In contrast, obtaining medical information from newspapers or television was not associated with that willingness (see Table 2).

Regarding what the participants consider important for their health management, *diet*, *exercise*, and *sleep* were all positive predictors in the univariate analysis of willingness to use PHRs (see Table 1). However—and strikingly—only exercise remained a strong predictor in the multivariate analysis (OR 1.57, *P*=.009) (see Table 2), while diet and sleep were discarded from the final model. It was reported that for type 2 diabetes patients a health behavior change in physical activity was less common than with a change in healthy diet, and that changing motivation for a healthy diet was higher than that for habitual physical activity [31]. Exercise is different from eating and sleeping behavior in that one has to actively make an effort to implement it [32], so patients who value exercise may have a greater tendency to actively pursue health. Similarly, people who collect medical information from books, magazines, and the internet may be more active in pursuing health than those who obtain information from more passive media like newspapers and television. This is consistent with the previous report that a health-related information-seeking personality was a significant predictor for willingness to undergo online treatment [21].

Interestingly, while *considering exercise important for their health management* was a positive predictive factor, *is willing to incorporate exercise into daily life for health management* was not a significant predictor in the multivariate analysis in this study (see Table 2). According to the Transtheoretical Model, health behavior change consists of sequential stages: precontemplation, contemplation, preparation, action, and maintenance [33]. Although questionnaire choices in this study do not precisely correspond to the stages of change in the Transtheoretical Model, people who *consider exercise important for their health management* are thought to be in one of the contemplation, preparation, action, or maintenance stages, while people who are *willing to incorporate exercise into daily life for health management* are thought to be in the action or maintenance stage. Those who *consider exercise important for their health management* but are not *willing to incorporate exercise into daily life for health management* are likely to be in the contemplation or preparation stage. The result that *considering exercise important* is a significant predictor of willingness to use PHRs—while *willing to incorporate exercise into daily life for health management* is not (see Table 2)—matches our previous report that diabetes patients who are in the contemplation or preparation stage of change in exercise are more willing to use ICT-based self-management tools [15]. Therefore, PHRs equipped with the function of stage-matched intervention for contemplation and preparation stages, such as understanding their own state, goal setting, or identification of barriers to behavior change, will lead the users to take action for health management, as stage-matched intervention was shown to be effective for improving glycemic control, hypertension control, and physical activity [34-37].

Because it was reported that health literacy was one of the key variables explaining a willingness to adopt a PHR [23], we expected that people who understand their disease well would be more willing to use a PHR. As expected, *understanding one’s diseases or symptoms well*, *understanding treatment methods well*, and *understanding how to deal with deterioration of health* were all positive predictive factors for willingness to use PHRs, while *entrusting doctors with disease and health-related matters* and *not paying much attention to one’s disease* were negative predictive factors in univariate analyses (see Table 1). In a multivariate analysis, however, *entrusting doctors with disease and health-related matters* was identified as a positive predictor, along with *understanding one’s diseases or symptoms well*. The reason for this seemingly paradoxical result is that multiple answers were allowed: people who answered *I entrust doctors with disease and health-related matters* were also likely to have chosen *I understand treatment methods well* and *I understand how to deal with deterioration of health*, both of which were strong positive predictors in univariate analyses.

While people who had high health literacy were found to be more willing to adopt a PHR [23], those with a low health literacy level might benefit most from a PHR. Plainly, educating people about the benefits of using PHRs will be important.

People who had used PHRs were asked about their usage. Although 94.3% (164/174) of them were willing to use PHRs (see Table 1), gaps between the contemplated and actual use were found (see Table 4). While many people wished to manage information using a PHR, they were not doing so (see Table 4). Future tasks include developing a PHR equipped with functions desired by the users, with the hope that the additions will help trigger usage. The most frequently managed information using PHRs was prescription details, with which there was no gap between the desired and actual function (see Table 4); this may be due to the fact that this study was done in pharmacies, where staff encouraged patients to use electronic prescription records as described above.

As noted, our results showed that 1302 out of 2307 participants (56.44%) expressed no willingness to use a PHR (see Table 1). Consistent with previous reports [38], the main reason for this disinclination was that using PHRs requires too much time and effort (see Table 6). It might be possible to capture the interest of such holdouts if there was a function that made information management quick and easy—perhaps something like auto-linking data on the order of inspection values between patients and medical institutions.

Other frequently mentioned reasons for unwillingness to use PHRs include no need being felt and concern over security. This points to the need for further efforts to inform people of the merits of using PHRs, as well as improvements in security.

This study suggested that the most prevalent information source regarding PHRs was the internet, if we eliminate the potential bias of the fact that the study was conducted in pharmacies where pharmacists encouraged patients to use electronic prescription records (see Table 5). This means that only people with high ICT literacy were able to get enough information about PHRs. Obviously, the visibility and usability of PHRs must be improved so that even citizens with low ICT literacy will find it easy to use.

### Limitations

There are several limitations to this study. First, because of the cross-sectional design of the study, the results do not reflect the long-term status of willingness to use PHRs, which might change drastically over time as the technology disseminates and comes into common use. Future tasks include updating the survey to follow the temporal changes in willingness.

Second, the survey was done in a geographically limited area: all the pharmacies were located in the Kanagawa Prefecture of Japan, considered part of the Greater Tokyo Area. Japan has the highest aging rate in the world with relatively high ICT literacy, so the results might not be valid for other regions with different age structures and levels of ICT literacy. Moreover, the eligibility criteria were determined by prescribed medications, which do not always correspond to a diagnosis. A previous study on claims-based algorithms for chronic conditions demonstrated that the sensitivity and specificity for identifying people with hypertension, diabetes, and dyslipidemia by using medication codes were 75.0% and 97.9%, 78.6% and 99.5%, and 34.6% and 97.2%, respectively [19]. Although the specificity is high, as expected, the sensitivity is relatively low, so the survey results cannot be generalized to the entire population with lifestyle-related diseases.

Third, because this was a self-administered questionnaire, some of the information such as the disease currently being treated may not be fully precise. As described above, 345 out of 2307 (14.95%) patients responded they were treated for neither hypertension, diabetes, nor dyslipidemia, even though all the participants were prescribed medication for at least one of the three diseases. Some of them may have been prescribed these medications to treat other diseases, but most of them presumably failed to self-report their diseases. Consistently, a previous report suggested that sensitivity of self-report questionnaires compared to pharmacy insurance claims was 92.4%, 82.6%, and 86.2% for hypertension, diabetes, and dyslipidemia, respectively [39]. Moreover, the intent of some questions may not have been fully understood or may have caused misunderstanding by the respondents, as it was not a structured interview. Importantly, although the concept of PHRs was explained both verbally and in writing as described in the Methods section, it is possible that the participants’ insufficient understanding or unfamiliarity with PHRs contributed to the lack of willingness, given that only 174 out of 2307 (7.54%) had experience using a PHR. Showing the participants actual examples of PHRs, such as health care management apps, prior to providing the questionnaire might help them better understand the concept of PHRs and properly determine the willingness to use PHRs.

Fourth, because the study was conducted at outlets of a dispensing pharmacy company whose staff encouraged patients to use electronic prescription records, there may have been biased results for some questions.

### Conclusions

We identified factors predicting a willingness to use a PHR by patients prescribed medication for lifestyle-related diseases. Patients with an active attitude toward health, such as those who consider exercise important for health management or who obtain medical information from books, magazines, or the internet, seem to be more willing to use a PHR. The main barriers to using a PHR were time and effort required and lack of perceived necessity, followed by concern over security. Analyzing these predictors and barriers will prove useful in identifying suitable candidates as PHR users and in developing more helpful tools for self-management by patients with lifestyle-related diseases.

## Appendix

Multimedia Appendix 1 The questionnaire used in this study.

Multimedia Appendix 2 The ownership rate of information and communication technology (ICT) devices (ie, personal computer, smartphone, and/or tablet) and willingness to use personal health records (PHRs) by age group.

## Abbreviations

eHealth: electronic health
EZR: Easy R
ICT: information and communication technology
OR: odds ratio
PHR: personal health record
