# Supplementary material for: Willingness of Patients Prescribed Medications for Lifestyle-Related Diseases to Use Personal Health Records: Questionnaire Study
Source: J Med Internet Res. 2020 May 28;22(5):e13866. doi: 10.2196/13866 (PMC7290452; doi:10.2196/13866)
Supplement: Multimedia Appendix 1 [file jmir_v22i5e13866_app1.docx]

◌ Single answer ☐Multiple answers

Basic data

| Sex | ◌ male ◌ female |
| --- | --- |
| Age | ◌ 20-29 ◌ 30-39 ◌ 40-49 ◌ 50-59 ◌ 60-69 ◌ 70-79 ◌ 80≤ |
| occupation | ◌ Business Owner, Executive ◌ Employee ◌ Self-employed person  ◌ Civil servant ◌ Househusband or housewife ◌ Part time worker  ◌ Student ◌ Unemployed ◌ Others(please specify) |

Medical history

| Q1 Are you currently being treated for any disease? (Please check all that apply)  ☐Hypertension ☐Diabetes ☐Dyslipidemia  ☐Angina pectoris/ Myocardial infarction ☐Arrhythmia ☐Kidney disease  ☐Hyperuricemia/Gout ☐Cerebrovascular disorder ☐Other (please specify) |
| --- |
| Q2.1 Have you ever been hospitalized?  ◌ Yes ⇒ please answer Q2.2  ◌ No |
| Q2.2 Most recent hospitalization.  Reason (name of disease or condition)?  Date of hospitalization? |
| Q3. Have you ever been diagnosed with metabolic syndrome?  ◌ Yes ◌ No ◌ Don’t know |

Awareness of health management

| Q4. What do you consider important for your health management? (Please check all that apply)  ☐Exercise ☐Diet ☐Sleep ☐Prescription drugs ☐Over-the-counter drugs  ☐Health food/supplements ☐Other (please specify) ☐None |
| --- |
| Q5. What is part of your daily life for managing health? (Please check all that apply)  ☐Exercise ☐Diet ☐Sleep ☐Prescription drugs ☐Over-the-counter drugs  ☐Health food/supplements ☐Other (please specify) ☐None |
| Q6. What is your source or sources of health information? (Please check all that apply)  ☐Doctors ☐Nurses ☐Pharmacists ☐Dieticians  ☐Other medical professionals (please specify) ☐Acquaintances ☐Family  ☐Books/Magazines ☐Newspapers ☐Television ☐Internet ☐Others (please specify) ☐None |
| Q7. How many medications are prescribed for you to take regularly, including oral administration and injection?  ◌ 1 ◌ 2 ◌ 3 ◌ 4 ◌ 5≤ |
| Q8. How often do you take medications?  ◌ Once a day ◌ Twice a day ◌ Three times a day ◌ 4 or more times a day  ◌ Once every few days ◌ Once a week ◌ Other (please specify) |
| Q9.1 Do you take medications as instructed by your doctor?  ◌ Yes  ◌ Not always ⇒ please answer Q9.2 and Q9.3 |
| Q9.2 How often do you fail to take medications?  ◌ About once a week ◌ About 2 or 3 times a week ◌ About 4 or 5 times a week  ◌ Almost every day |
| Q9.3 What are the reasons for failing to take medications? (Please check all that apply)  ☐I forget to take them ☐Too frequent dosing ☐Too many medications  ☐Usage is too complicated ☐I cannot feel the effect ☐Concern over side effects  ☐I don’t understand the purpose of taking them ☐Medications are too expensive  ☐Other (please specify) |
| Q10. How well do you understand your diseases? (Please check all that apply)  ☐I understand my diseases/symptoms well.  ☐I understand treatment methods well.  ☐I understand how to deal with deterioration of health.  ☐I entrust my doctor with my disease and health-related matters.  ☐I do not pay much attention to my disease. |
| Q11. How often do you visit a pharmacy?  ◌ Twice a month or more ◌ About once a month ◌ Once every few months  ◌ About once in six months |
| Q12. 1 Do you use the family pharmacist system?  ◌ Yes  ◌ No ⇒ please answer Q12.2  ◌ Don’t know of it |
| Q12.2 What is the reason for not using the family pharmacist system? (Please check all that apply)  ☐It costs too much  ☐I don’t feel I would get enough service  ☐I don’t feel the need  ☐I am satisfied with current service  ☐I don’t know the system  ☐I use other pharmacies  ☐Other (please specify) |
| Q13. Are you using Okusuri Techo Plus (an electronic prescription record application for smartphones and personal computers developed by Nihon Chouzai)  ◌ I am currently using it  ◌ I have used it before  ◌ I know of it but have never used it  ◌ I do not know it |

Personal health record (PHR)

| Q14. What kind of information terminal do you have? (Please check all that apply)  ☐Personal computer ☐Smartphone ☐Basic cell phone ☐Tablet device  ☐Other (please specify) ☐None |
| --- |
| Q15.1 Have you ever used PHR?  ◌ I am using it ⇒ please answer Q15.2-6  ◌ I have used it before ⇒ please answer Q15.2-6  ◌ I know of it but have never used it  ◌ I do not know it |
| Q15.2 What type of PHR applications do you use/have you used? (Please check all that apply)  ☐Electronic prescription record ☐Weight management  ☐Blood pressure management ☐Blood sugar management  ☐Activity amount recorders ☐Menstruation prediction  ☐Information of sleep ☐Others (please specify) |
| Q15.3 What is the source of your information about PHRs? (Please check all that apply)  ☐Hospital staff ☐Pharmacy staff ☐Acquaintance ☐Family ☐Television  ☐Newspapers ☐Internet ☐Books, Magazines ☐Poster ☐Other (please specify) |
| Q15.4 What type of information would you like to manage by PHR? (Please check all that apply)  ☐Test results at medical institution ☐Home blood pressure values and body weight, etc  ☐Meal content ☐Record of immunization ☐Medication adherence  ☐Medication prescription details ☐Record of the date of hospital visit  ☐Surgery record ☐Medical history ☐Medical history of the family  ☐Others (please specify) |
| Q15.5 What type of information do you manage by PHR? (Please check all that apply)  ☐Test results at medical institution ☐Home blood pressure values and body weight, etc  ☐Meal content ☐Record of immunization ☐Medication adherence  ☐Medication prescription details ☐Record of the date of hospital visit  ☐Surgery record ☐Medical history ☐Medical history of the family  ☐Other (please specify) |
| Q15.6 What function of PHR would you like for health management? (Please check all that apply)  ☐Medical data management  ☐Automatic recording of measurement data (e.g. blood pressure, body weight)  ☐Printing medical information ☐Automatic reading of printed test results  ☐Information sharing with medical staffs ☐Reminder for self-measurement  ☐Reminder to take medication ☐Manage allergy information  ☐Emergency contact details ☐Other (please specify) ☐None |
| Q16.1 Would you like to use PHR in the future, whether or not you currently use it?  ◌ I would like to use it  ◌ I will probably use it  ◌ I probably will not use it ⇒ please answer Q16.2  ◌ I will not use it ⇒ please answer Q16.2 |
| Q16.2 What is the reason for not using PHR? (Please check all that apply)  ☐It will take more time and effort.  ☐I feel no need to use PHR.  ☐I am concerned about security.  ☐Other (please specify) |
