# Supplementary material for: Willingness of Patients Prescribed Medications for Lifestyle-Related Diseases to Use Personal Health Records: Questionnaire Study
Source: J Med Internet Res. 2020 May 28;22(5):e13866. doi: 10.2196/13866 (PMC7290452; doi:10.2196/13866)
Supplement: Multimedia Appendix 2 [file jmir_v22i5e13866_app2.docx]

**Multimedia Appendix 2.** The ownership rate of ICT devices (personal computer and/or smartphone and/or tablet) and willingness to use PHRs by the age groups. (n=2290)


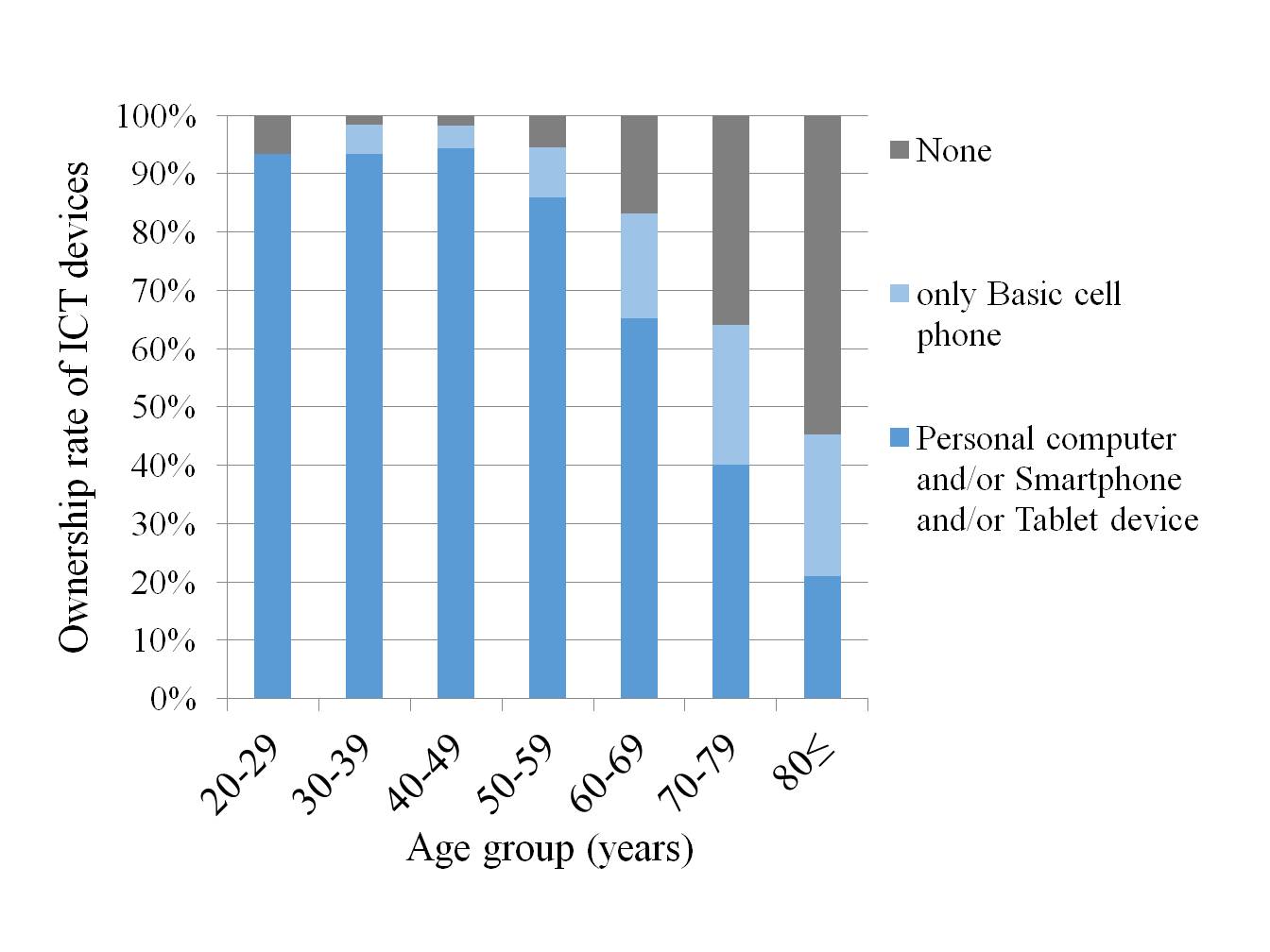


| Age group  (n=2290) | Ownership rate of personal computers and/or smartphone and/or tablet , n (%)  (n=2200) | | Willingness to use PHRs (n=2108), n(%) | |
| --- | --- | --- | --- | --- |
| <60 years old (n=654) | Yes | 580 (88.7) | Yes | 345 (59.5) |
|  |  |  | No | 225 (38.8) |
|  | No | 71 (10.9) | Yes | 18 (25) |
|  |  |  | No | 49 (69) |
| ≥60 years old (n=1636) | Yes | 718 (43.89) | Yes | 345 (48.1) |
|  |  |  | No | 348 (48.5) |
|  | No | 831 (50.79) | Yes | 136 (16.4) |
|  |  |  | No | 642 (77.3) |
